# A Series of Biliary Tract Cancer With Coexistent Non-biliary Second Malignancy From Sub-Himalayan Region of India

**DOI:** 10.7759/cureus.13415

**Published:** 2021-02-18

**Authors:** Deepak Rajput, Amit Gupta, Sweety Gupta, Shashank Kumar

**Affiliations:** 1 Department of Surgery, All India Institute of Medical Sciences, Rishikesh, Rishikesh, IND; 2 Department of Radiation Oncology, All India Institute of Medical Sciences, Rishikesh, Rishikesh, IND

**Keywords:** biliary tract cancer, dual malignancy, incidental, non-biliary, second primary, synchronous

## Abstract

Background

When researching female patients with breast or ovarian neoplasms, our research will sensitize oncologists to the prevalence of biliary tract cancers such that early cancers are not overlooked. Depending on different inherited, environmental, and iatrogenic risk factors, patients diagnosed with cancer have a risk of harboring another de novo malignancy. The additional primary identification of late has increased mainly due to progress in both diagnosis and treatment modalities, improvement in life expectancy, and understanding.

Methods

This is a descriptive study of retrospectively collected data from health records over 15 months, of patients who had biliary tract cancer and incidentally detected coexisting second non-biliary malignancy, from July 2018 to September 2019 at a tertiary care hospital. Details such as age, sex, smoking history, family history, occupation, body mass index (BMI), the organ involved, levels of tumor markers, treatment, and outcome were recorded.

Results

Six consecutive patients with biliary tract cancer presented during this duration and incidentally detected the second primary was ovarian cancer in three (50%) patients, breast carcinoma in two (33%) patients, and urinary bladder carcinoma in the remaining one patient (17%). The median age at diagnosis was 52.5 years with a range of 40-65 years. All patients were females (100%), non-smokers, homemaker, and without any history of cancer in family members. Only two patients who had a resectable disease were alive at one year’s follow-up.

Conclusion

The mechanisms of carcinogenesis in multiple primary malignancies are mainly genetic, epigenetics, and immunological. Prognosis, as well as the intent of treatment, depends on the respective stages of the two malignancies. In our study, most of the patients were in an advanced stage that demanded palliative care.

## Introduction

It is not unusual to develop a second malignancy de novo in a patient with a confirmed malignant tumor. Billroth first identified the multiple primary malignant neoplasms (MPMN) phenomenon at the end of the 19th century [1], and many cases of double or even triple primary malignant neoplasms have since been recorded. Depending on whether the analysis is antemortem or postmortem [2,3], the reported incidence varies from 0.73%-11.3%. Owing to a rise in the number of elderly patients and advances in diagnostic methods, metachronous primary malignancies are becoming increasingly common. Synchronous primary malignancies, however, are also rare. Also, new diagnostic technologies, such as positron emission tomography and computed tomography (PET-CT), image-guided tissue biopsy, and immunohistochemistry, the use of more rigorous surveillance and screening for second cancers, have also significantly contributed to increasing the detection of multiple malignancies. Data regarding treatment and its outcome in such cases are sparse. We report our experience of six cases of a combination of synchronous detected non-biliary malignancies with biliary tract cancers, which, to the best of our knowledge, has never been previously reported in the literature from any part of the world.

## Materials and methods

Warren and Gates first presented the criterion used for diagnosing multiple primary cancers (Table 1).

**Table 1:** Warren and Gates' criteria for the diagnosis of multiple primary malignancies

No.	Criteria
1.	Each of the tumors must be malignancy confirmed by histology.
2.	Each must be geographically separate and distinct. The lesions should be separated by normal mucosa.
3.	The probability of one being the metastasis of the other must be excluded.

This is a descriptive study of retrospectively collected data of patients with the diagnosis of biliary tract cancers and incidentally detected coexisting second malignancies over 15 months from July 2018 to September 2019 who presented in the Hepato-Pancreato-Biliary (HPB) division of the Department of General Surgery at a tertiary care hospital in the sub-Himalayan region of Uttarakhand, India. The inclusion criteria of patients in this study were the presence of one neoplastic location in the biliary tree and a second elsewhere in the body, that confirmed by histopathological examination, with distinct histopathology in the two locations. Contrast-enhanced computed tomography (CECT) was the imaging modality used to stage the disease. Various details such as age at presentation, sex, history of smoking, family history, occupation, body mass index (BMI), the organ involved, levels of tumor markers [carcinoembryonic antigen (CEA), carbohydrate antigen (CA)19-9, CA125, lactate dehydrogenase (LDH), alpha-fetoprotein (AFP)], treatment, and outcome were recorded.

Case 1

**Figure 1:**
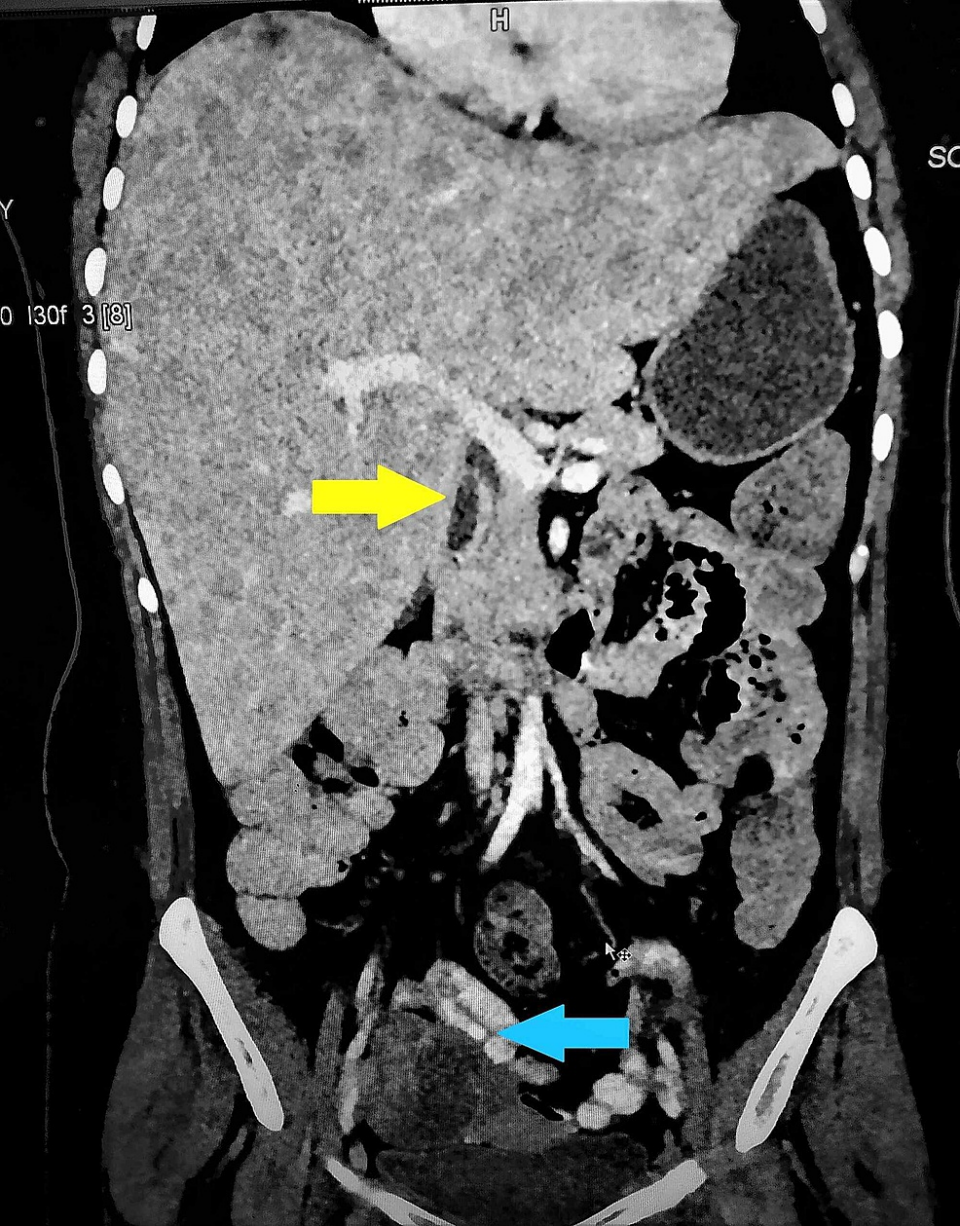
Coronal section of the abdominal tomography scan showing gall bladder thickening (yellow arrow) and coexistent right adnexal mass (blue arrow).

A 40-year-old lady presented to the outpatient clinic with complaints of pain right upper abdomen, associated vomiting on and off, and loss of appetite for the past 15 days. She had no bladder or bowel complaints with normal menstrual cycles and three living children. General physical examination was unremarkable. Abdominal examination revealed enlarged non-tender liver with free fluid in the peritoneal cavity. Ultrasonography (USG) abdomen showed multiple gall stones and right adnexal mass with suspected liver metastasis. Tumor markers CA19-9 (27 U/mL) and CEA (4.07 ng/mL) were within the normal range, but CA125 (56.6 U/mL), LDH (487 U/L), and AFP (277 ng/mL) were elevated. CECT abdomen and pelvis showed a neoplastic growth of gall bladder with contiguous hepatic involvement and multiple hepatic metastases. A solid cystic right adnexal mass impinging on the bladder and abutting adjacent bowel loops was also seen (Figure 1).

The patient was planned for an ultrasound (USG) guided fine-needle aspiration cytology (FNAC) from liver space-occupying lesion and the right adnexal mass. The lesion in the liver revealed metastatic adenocarcinoma, while the right adnexal mass showed clusters of atypical cells suggestive of malignancy. Given advanced stage and poor prognosis, she was referred to the medical oncology department for palliative chemotherapy (Capecitabine).

Case 2

**Figure 2:**
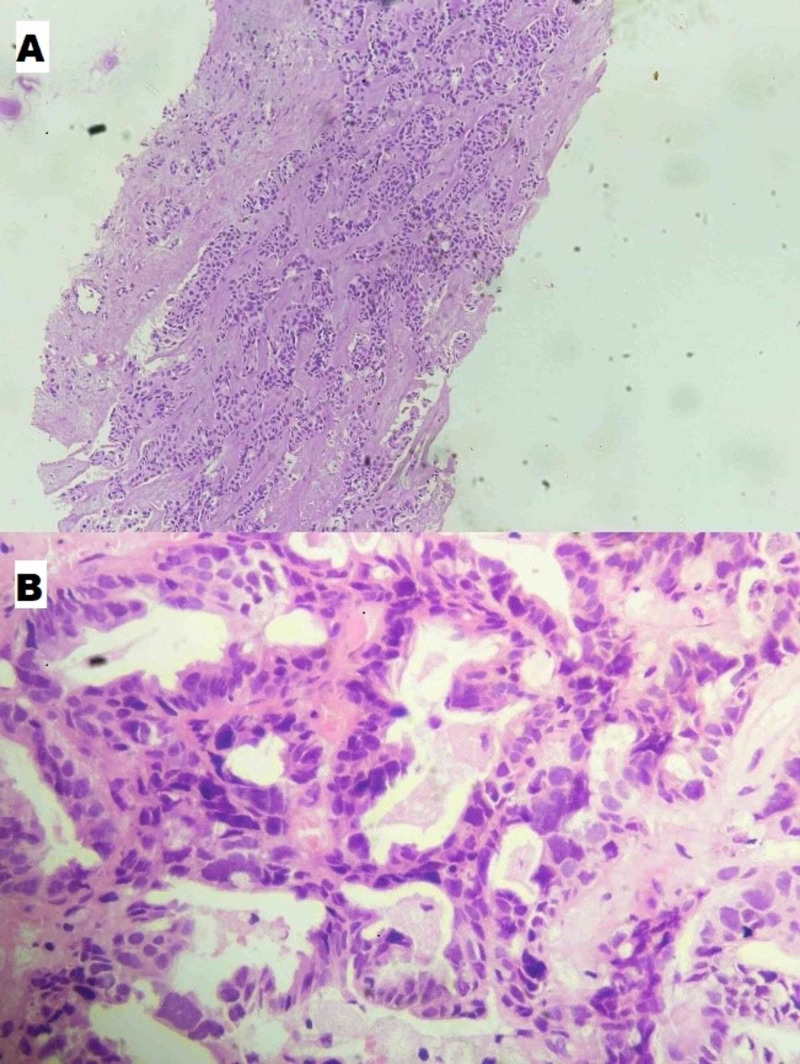
Microphotograph 10x view showing (A) infiltration by a tumor arranged in nests and tubules in liver metastasis and 40x view demonstrating (B) tumor cells arranged in cribriform architecture with pleomorphic cells in carcinoma ovary.

A 50-year-old postmenopausal lady, known case of hypertension on irregular medication for the same, presented in the outpatient department with complaints of on-and-off right-sided upper abdominal pain for the past one year and associated one-week history of yellowish discoloration of eyes at the onset of illness, which resolved on its own. There was a history of significant weight loss and loss of appetite. Bladder and bowel habits were normal. General physical examination was unremarkable. On abdominal examination, a non-tender hard mass of size 4x4 cm was palpable in right hypochondrium, moving with respiration, and the superior margin was not felt separate from the enlarged liver. Laboratory tumor markers CA19-9, CA125, and CEA were elevated. Liver function tests were normal. CECT abdomen and pelvis showed a large infiltrating mass measuring approx. 6.3x4.4x4.9 cm arising from the gall bladder fundus and infiltrating adjacent IVb and V segments of the liver. There were multiple heterogeneously enhancing lesions seen in both lobes of the liver (largest 25x24 mm in segment IVb) suggestive of metastasis. There was also a solid enhancing mass of size noted in the right adnexa abutting uterine fundus. The right ovary could not be visualized separately from the lesion. The patient underwent a USG guided FNAC from the liver and right adnexal lesions which revealed metastatic adenocarcinoma and papillary adenocarcinoma respectively (Figures 2A, 2B).

The attendants were explained about the prognosis, following which they consented to palliative chemotherapy.

Case 3

**Figure 3:**
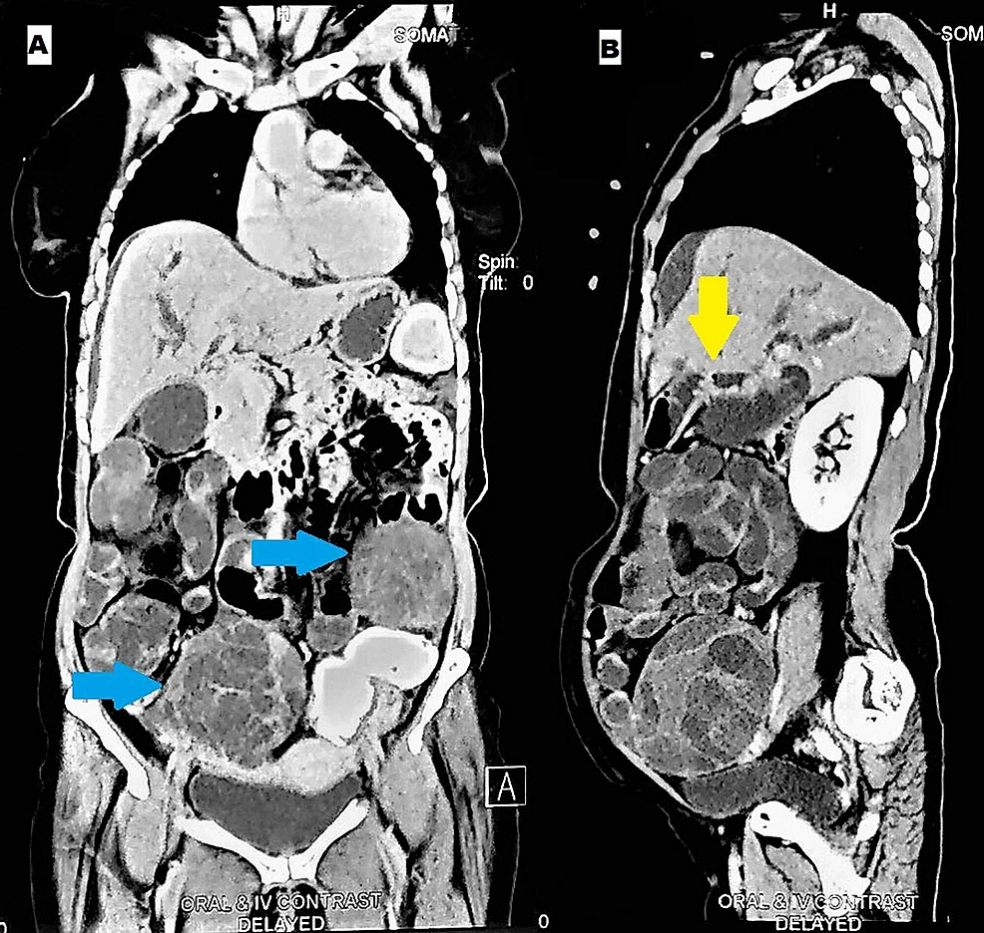
Abdominal computed tomography scan in delayed phase showing coexistent proximal bile duct cholangiocarcinoma (yellow arrow) and bilateral adnexal masses (blue arrows) in (A) coronal and (B) sagittal sections.

A 64-year-old postmenopausal lady presented to the outpatient department with complaints of pain upper abdomen on and off, yellowish discoloration of sclera, and itching all over the body for the past three months. The patient had a history of significant weight loss and loss of appetite with no alteration in bladder and bowel habits. The patient had a thin build and was icteric on general physical examination with poor performance status. On abdominal examination, large non-tender intra-abdominal intraperitoneal masses with irregular surface and margins were palpable over the right lumbar, right iliac fossa, hypogastrium, left iliac fossa, and left lumbar regions. They had variable consistency and the lower edge disappeared under pubic symphysis. Ultrasound abdomen showed bilateral adnexal masses with gall stones. CECT abdomen and pelvis revealed cholelithiasis and an ill-defined suspicious mass in proximal bile duct with enhancing wall thickening for a length of 2.8 cm reaching superiorly up to the primary confluence. A note was also made of large bilateral adnexal masses abutting the anterior abdominal wall with loss of fat planes with pelvic small bowel loops (Figure 3).

The liver function test showed total bilirubin of 289 µmol/L with the level of the direct component being 170 µmol/L. Tumor markers CA19-9 (160.7 U/mL) and CA125 (104.8 U/mL) were elevated and CEA (3.49 ng/mL) was normal. Because of poor performance status, the patient was planned for percutaneous transhepatic biliary drainage (PTBD) to alleviate jaundice and USG guided FNAC from adnexal masses that showed features suspicious of malignancy. The patient was planned for palliative chemotherapy after the decrease of total bilirubin level to at least 51 µmol/L.

Case 4

**Figure 4:**
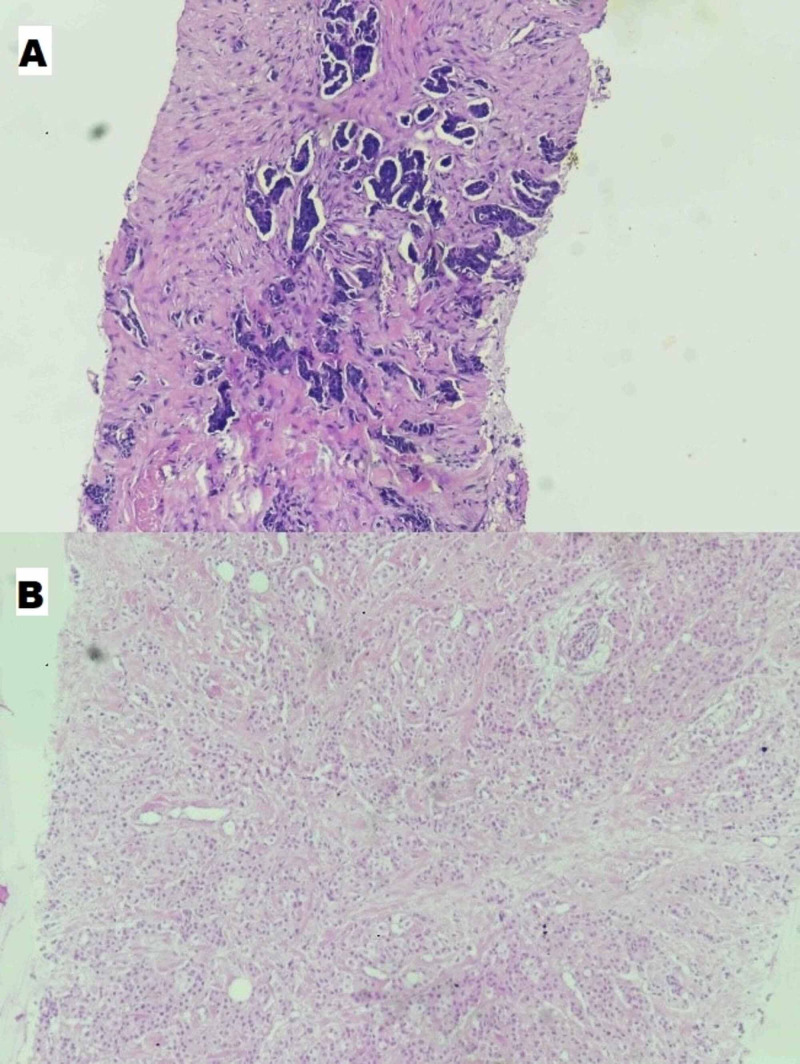
Microphotograph 10x view showing (A) tumor cells lying in small clusters and lying dyscohesively in omental deposits and (B) tumor cells arranged in sheets and tubules in invasive ductal carcinoma breast.

A 53-year-old postmenopausal lady presented in the outpatient department with insidious onset, non-progressive upper abdominal pain for the past six months associated with yellowish discoloration of eyes. There was a recent onset history of fever and vomiting. History of significant weight loss and loss of appetite was present. She also noticed a lump in her left breast for the past three months, which was painless, non-progressive with no nipple discharge and skin changes. General physical examination revealed pallor and no axillary lymphadenopathy. The abdominal examination was unremarkable. A hard, non-tender mobile lump of size 4x4 cm was palpable in the upper outer quadrant of the left breast with a normal nipple-areola complex. Laboratory reports revealed anemia, leucocytosis, and obstructive jaundice with a deranged coagulation profile. USG abdomen showed an ill-defined heteroechoic lesion sized 3x2.6x3.7 cm in gall bladder fundus and body region with ill-defined fat planes with adjacent liver parenchyma. The liver also showed a well-defined hypoechoic lesion 1.4x1.5 cm adjacent to gall bladder fossa likely metastatic. The common bile duct (CBD) was dilated proximally measuring around 14.8 mm in size with central intrahepatic biliary radicle dilatation (IHBRD). Few heterogeneously enhancing nodular omental deposits were also seen that revealed metastatic adenocarcinoma on biopsy (Figure 4A).

With the working diagnosis of obstructive jaundice and cholangitis, the patient was planned for PTBD after starting intravenous antibiotics and normalization of international normalized ratio (INR) by fresh frozen plasma (FFP) transfusion. A trucut biopsy done from the lump in the left breast revealed an infiltrating duct carcinoma (Figure 4B). The advanced stage of the disease, grave prognosis of the patient, and a high chance of mortality were explained to the patient’s attendants who henceforth requested a discharge for home care. However, the patient was readmitted to the emergency department after four months with altered sensorium, blood pressure 70/40 mm Hg, decreased urine output, and bile leak from the PTBD site. Despite resuscitative measures, the patient could not be salvaged.

Case 5

A 65-year-old postmenopausal lady presented in the breast clinic with a painless lump in her right breast and greenish nipple discharge for the past two months. She also gave a history of yellowish discoloration of the sclera two months back, which lasted for a week and was associated with pain right upper abdomen that got relieved on taking medications. Associated history of significant weight loss and loss of appetite was present. She had a history of vaginal hysterectomy three years back. On local examination, a firm-to-hard non-tender mobile lump of size 2x3 cm in the lower inner quadrant of the right breast with normal-appearing nipple-areola complex and a single enlarged central group axillary lymph node were palpable. General physical examination and abdominal examination were unremarkable. Mammography of the affected breast revealed a BIRADS-4 lesion that showed features suspicious of malignancy on FNAC. Ultrasound abdomen done for the metastatic workup showed diffusely thickened gall bladder wall in the region of body and fundus. CECT scan of the abdomen confirmed an ill-defined heterogeneously enhancing lesion measuring 2.6x2.5x2.7 cm in the body region of the gall bladder, abutting the first part of the duodenum medially and few heterogeneously enhancing pericholedochal lymph nodes. The patient underwent a right modified radical mastectomy and radical cholecystectomy and was planned for adjuvant chemotherapy.

Case 6

**Figure 5:**
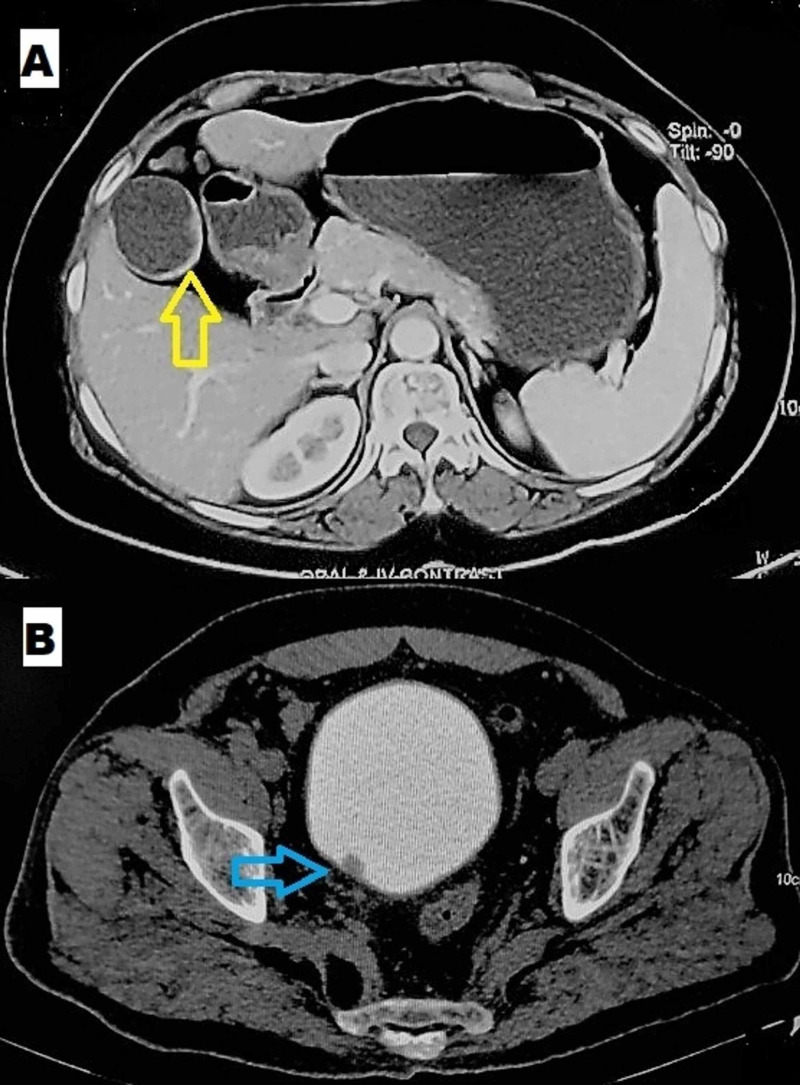
Axial section of the abdominal tomography scan showing (A) gall bladder thickening depicted by the yellow arrow and (B) blue arrow pointed towards polypoidal lesion in the urinary bladder.

A 63-year-old female came to the surgical outpatient department with complaints of lower abdominal dull aching pain that was associated with increased frequency of urination and dysuria over the past month. She also had an episode of painless hematuria one month prior and gave the history of significant loss of weight and appetite. Ultrasound abdomen revealed an asymmetric gall bladder thickening in the body region and suspicious mass in the urinary bladder. CECT scan of the abdomen and pelvis demonstrated an irregular thickening over the body region of the gall bladder (Figure 5A) with maintained fat planes with the first part of the duodenum and a polypoidal lesion in the urinary bladder (Figure 5B).

The levels of tumor markers CA19-9, CEA, and CA125 were in the normal range. She underwent radical cholecystectomy with transurethral resection of bladder tumor (TURBT) and was planned for adjuvant chemotherapy.

## Results

**Figure 6:**
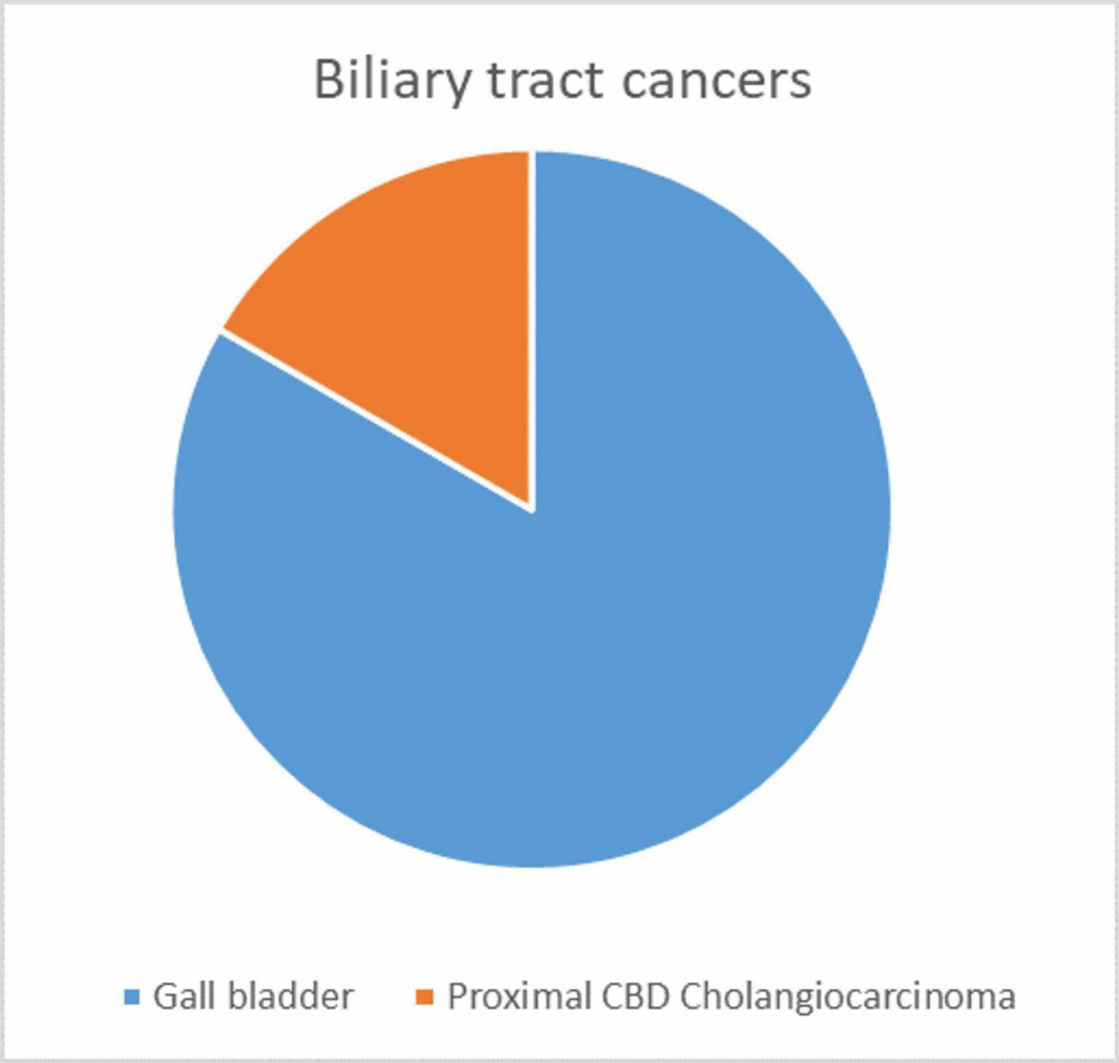
Pie chart illustrating the organ-wise distribution of biliary tract cancers in our study CBD - common bile duct

**Figure 7:**
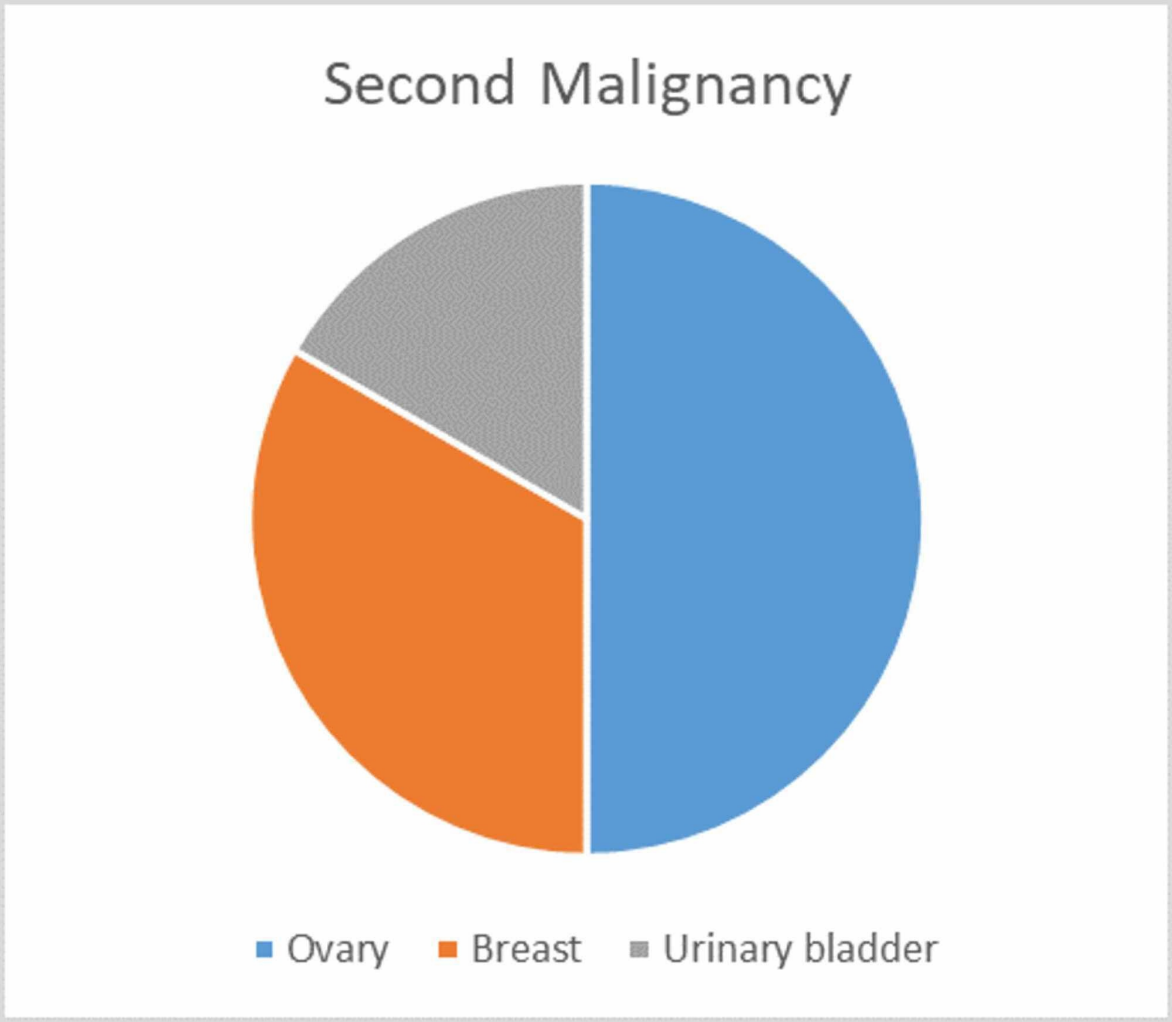
Pie chart illustrating an organ-wise distribution of the second malignancy in our study

Six patients presented with a synchronous double malignancy associated with biliary tract cancer during the study period. Four patients had no specific symptoms related to the second neoplasm, which was detected incidentally during evaluation of the first primary. The first primary was gall bladder cancer in five patients and proximal bile duct cholangiocarcinoma in one patient (Figure 6), while the second neoplasm was ovarian cancer in three patients, breast cancer in two patients, and urinary bladder cancer in one patient (Figure 7).

Two patients presented with obstructive jaundice and cholangitis at admission while a history of jaundice was elicited from two more patients. Two patients were offered palliative chemotherapy due to the advanced stage of the disease while the two that had cholangitis at presentation underwent an urgent ultrasound-guided PTBD. Out of the remaining two patients that were found to have resectable disease, one underwent transurethral resection of bladder tumor (TURBT) and radical cholecystectomy while the other a modified radical mastectomy (MRM) and radical cholecystectomy (Table 2).

**Table 2:** Summary of biliary tract cancer and synchronous non-biliary malignancy

Age/Sex	Biliary tract cancer	Treatment	Non-biliary malignancy	Treatment	Outcome
40/F	Gall bladder cancer with liver metastasis	Palliative chemotherapy	Right adnexal mass	Palliative chemotherapy	Expired in three months
50/F	Gall bladder cancer with liver metastasis and periportal lymphadenopathy	Palliative chemotherapy	Right adnexal mass	Palliative chemotherapy	Expired in six months
64/F	Proximal bile duct cholangiocarcinoma with obstructive jaundice and cholangitis	Percutaneous transhepatic biliary drainage	Bilateral adnexal mass	Planned for chemotherapy	Expired in one month
53/F	Gall bladder cancer with obstructive jaundice and cholangitis	Percutaneous transhepatic biliary drainage	Carcinoma left breast	Planned for chemotherapy	Expired in four months
65/F	Gall bladder cancer (Early)	Radical cholecystectomy	Carcinoma right breast	Modified radical mastectomy	Alive and on adjuvant chemotherapy
63/F	Gall bladder cancer (Early)	Radical cholecystectomy	Carcinoma urinary bladder	Transurethral resection of bladder tumor	Alive and on adjuvant chemotherapy

Biopsy from tissue samples after radical cholecystectomy, TURBT, and MRM showed well-differentiated adenocarcinoma, low-grade transitional cell carcinoma without any muscle invasion, and invasive ductal carcinoma respectively.

The median age at diagnosis was 52.5 years with a range of 40-65 years. The four patients who had unresectable disease died over a period ranging from one to six months. The remaining two patients were alive at one year’s follow-up. All patients were females (100%), non-smokers, homemakers without exposure to any type of industrial hazard, and any history of cancer-related deaths in family members. The tumor markers CEA and CA19-9 were elevated in biliary tract cancers with cholangitis, the extent of spread to adjoining structures and lymph nodes. CA125 was elevated in patients with coexistent adnexal masses (Table 3).

**Table 3:** Summary of biliary tract cancer and synchronous non-biliary malignancy (continued) BMI - body mass index; CEA - carcinoembryonic antigen; CA - carbohydrate antigen

Age/Sex	Smoking history	Family history of malignancy	BMI (kg/m^2^)	CEA (ng/mL)	CA19-9 (U/mL)	CA125 (U/mL)
40/F	no	no	26.3	4.07	27	56.6
50/F	no	no	24.1	38.53	9137	59.2
64/F	no	no	21.6	3.49	160.7	104.8
53/F	no	no	19.1	3.53	157.3	27.3
65/F	no	no	24.3	32.5	214.8	21.7
63/F	no	no	21.9	1.49	21.05	14

## Discussion

In-person diagnosis of synchronous primary cancers is uncommon and complicated [4]. The signs of the first neoplasm will obscure the second malignancy and the diagnosis can be complicated by the likelihood of local or distant recurrence of first cancer. They should not be ignored as metastatic diseases if such tumors are accidentally detected. To rule out the rare possibility of a second primary, any irregular metastasis site should be thoroughly examined. Baseline PET-CT can assist in the diagnosis of multiple tumors of this type and, in some cases, in the treatment plan [5]. Nonetheless, in conjunction with standard clinical and radiological requirements, PET appears to be a useful diagnostic tool, especially when considering older comorbid patients at high surgical risk.

Synchronous malignancies diagnosed with cancers of the biliary tract have rarely been reported from the Indian subcontinent; hence, maintaining a high index of suspicion while examining such lesions is necessary for the clinician as well as the pathologist. Four major factors may be linked to the development of several primary malignancies: intrinsic factors, extrinsic factors, genetic factors, and therapeutic factors [6,7]. Susceptibility, immune status, and endocrine and embryonic growth include intrinsic influences. Exposure can occur exogenously when these agents are present in food, air, or water, as well as endogenously when they are metabolic or pathophysiological products, such as inflammation. Besides, major carcinogenic variables are smoking and alcohol, and poor physical activity. Various deoxyribonucleic acid (DNA) microsatellite instability (MSI)-related syndromes, such as Lynch I and II syndromes, are associated with the development of multiple primary tumors in various organs. The risk of developing malignancy of the biliary tract in Lynch syndrome is 2%-4% [8]. There is an increased risk of early-onset breast and ovarian cancer in members of families with breast cancer (BRCA) gene mutations [9]. Kimura et al. found germ-line p53 mutation in a patient with multiple primary cancers [10].

It would be important to investigate the replication errors (RER) at microsatellite loci on different chromosomes to show whether a genetic defect affecting the DNA repair and replication mechanism is present. If the RER phenotype is positive, then the development of several primary cancers is advantageous. Testing the RER phenotype in primary cancer patients would be acceptable to identify the highest-risk patients among them [11]. For the differential diagnosis of carcinomas arising from different locations, the combination of cytokeratin (CK) expression CK7 and CK20 is often known to be used.

A review of literature done showed publication of a short series of seven cases of the synchronous gall bladder and bile duct cancer [12], which could be due to anomalous pancreaticobiliary ductal junction (APBDJ), de novo multifocal origin, or as part of a field change. APBDJ also suggests a probable association between ampullary and pancreatic neoplasms [13]. Case reports of hepatocellular carcinoma (HCC) and intrahepatic cholangiocarcinoma (ICC) have been reported suggesting field carcinogenesis [14]. To date, ICC synchronous with HCC [15], ampullary carcinoid [16], lymphoma [17], lung squamous cell carcinoma [18], lymphoepithelioma [19], and renal cell carcinoma [20] have been reported, while few cases have been reported synchronous with thyroid carcinoma. Case reports of the synchronous primary gall bladder and rectal cancers have been published in the past [8]. Some studies suggest that their simultaneous occurrence is mainly due to gallstones and not to the genetic or hereditary risk. In reality, the fecal excretion of bile acids is increased in patients suffering from colorectal cancer and those who have undergone cholecystectomy.

A strong association between biliary tract cancers and ovarian/breast cancer may be due to similar risk factors such as obesity as well as high fat diet and fat intake >38% of daily calories but this was not seen in our study. Association between urinary bladder carcinoma and gall bladder cancer could be sporadic. Screening for second malignancies is an attractive option, but the optimal screening modalities with cost-effectiveness in mind elude us for most cancers.

With a very poor prognosis, biliary tract cancer (BTC) is still a terminal illness. A major challenging issue in the diagnosis and management of BTC is the lack of appropriate biomarkers for early diagnosis and successful therapeutic goals. Due to the clinically silent and asymptomatic characteristics of the tumor, most patients are diagnosed at an already advanced stage allowing only for a palliative therapeutic approach.

Limitations

The present report contains retrospective data from health records from which association but not causation can be derived. The causes of de novo synchronous malignancies are not yet apparent and deserve to be thoroughly investigated. While risk factors, such as smoking, alcohol intake, and unhealthy lifestyles, can be reduced, non-modifiable risk factors, such as genetic vulnerability, cannot be controlled through controlling immune deficiencies.

## Conclusions

This research reveals an especially unusual clinical image of synchronous dual malignancy seen in female patients, so the need for a good comprehensive history and thorough breast and pelvic examination can never be overemphasized along with clinical staging for any primary malignancy so that, if present, another de novo malignancy does not go unnoticed. In the case of synchronous double malignancy, treatment methods will rely on the first treatment of the malignancy that is more advanced or often both may be handled concurrently by interdisciplinary coordination and at the same time seeking to prevent negative effects on the overall outcome due to increased toxicity or related pharmacological interaction.
